# Reconstruction strategies for extensive bone defects following resection of primary malignant tumors in the distal radius

**DOI:** 10.3389/fonc.2025.1644295

**Published:** 2025-10-20

**Authors:** Qing-He Guo, Wei Wang, Zheng-Ming Yang, Yun-Jing Qiu, Jia-Dan Wu, Ke-Yi Wang, Hui-Min Tao, Zhao-Ming Ye

**Affiliations:** ^1^ Department of Orthopedics, The Second Affiliated Hospital of Zhejiang University, Hangzhou, Zhejiang, China; ^2^ Orthopedics Research Institute of Zhejiang University, Hangzhou, Zhejiang, China; ^3^ Department of orthopedics, Yunhe County People’s Hospital, Lishui, Zhejiang, China; ^4^ School of Nursing and Midwifery, University of Technology Sydney, NSW, Australia

**Keywords:** bone defect, distal radius, malignancy, resection, reconstruction

## Abstract

**Background:**

This study aimed to evaluate surgical resection techniques for primary malignant tumors of the distal radius and to examine the reconstruction strategies employed for resulting large bone defects.

**Methods:**

A retrospective analysis was conducted on 11 cases involving the resection and reconstruction of primary malignant tumors in the distal radius. Complete medical records and follow-up data from December 2013 to December 2022 were reviewed. Oncological outcomes were assessed, with specific focus on the extent of tumor invasion, surgical resection techniques, and reconstruction strategies.

**Results:**

The follow-up period ranged from 5 to 118 months, with a mean duration of 41.6 months. At the final follow-up, local recurrence was observed in three patients, six died due to disease progression, and five were alive. For tumors confined to the distal radius, resection of the distal radius follow by joint-preserving reconstruction using either autologous fibular grafts or 3D-printed prostheses. For tumors involving the distal radius, proximal carpal bones, and/or distal ulna, wrist arthrodesis are chose using either autologous fibular grafts or ipsilateral ulnar transposition with arthrodesis For tumors involving more than half of the distal radius, reconstruction strategies include ulnar-only fixation or wrist arthrodesis with ipsilateral ulnar transposition.

**Conclusions:**

Primary malignant tumors of the distal radius are rare and associated with a poor prognosis. Selection of resection and reconstruction techniques is primarily influenced by the extent of tumor invasion, with joint-preserving strategies feasible in selected cases and arthrodesis required for more extensive bone involvement.

## 1 Introduction

Primary malignant tumors of the distal radius are rare, with giant cell tumors more frequently reported in the literature. Large segmental resection of the distal radius is the main treatment approach, and achieving a wide surgical margin is essential for malignant tumors. In certain cases, resection of the distal radius alone may be inadequate, necessitating removal of the proximal carpal bones and/or adjacent segments of the ulna, depending on the extent of tumor involvement. The choice of reconstruction techniques is influenced by variability in resection approaches. As primary malignant tumors in the distal radius are rare, only a limited number of case reports pertaining to osteosarcoma, chondrosarcoma, and angiosarcoma are available in the literature (1–4). The scarcity of cases has led to a lack of consensus on optimal resection and reconstruction strategies for extensive segmental defects. Available reconstruction options include joint-preserving techniques such as autologous fibular grafts, allograft distal radius bone transplantation, and the use of 3D-printed prostheses. When joint preservation is not feasible, wrist arthrodesis may be performed using autologous iliac bone grafts, autologous fibular grafts, 3D-printed prostheses, or ipsilateral ulnar transposition with arthrodesis (5).

Given the limited literature on primary malignant tumors in the distal radius, a comprehensive analysis of resection methods and reconstruction techniques remains lacking. In this study, 11 cases of primary malignant tumors in the distal radius were retrospectively analyzed using complete medical records and follow-up data from a hospital. Oncological outcomes were summarized, and the extent of tumor involvement, resection methods, and the choice of reconstruction strategies were analyzed.

## 2 Methods

### 2.1 General information

#### 2.1.1 Inclusion and exclusion criteria

##### 2.1.1.1 Inclusion criteria

The following cases were included in the study: (1) primary malignant tumor of the distal radius; (2) No metastasis occurred during treatment; (3) Surgical treatment was performed; (4) availability of complete medical records and follow-up data.

##### 2.1.1.2 Exclusion criteria

The following cases were excluded: (1) giant cell tumors; (2) metastatic tumors; (3) tumor invasion of vital blood vessels and nerves; and (4) lost to follow-up cases.

#### 2.1.2 Study sample

A retrospective analysis was conducted on 11 patients with primary malignant tumors in the distal radius treated at a hospital between December 2013 and December 2022. This sample consisted of 6 males and 5 females, aged 7 to 74 years (mean age: 43.2 years). Diagnoses included osteosarcoma (*n* = 4 cases), undifferentiated sarcoma (*n =* 2), angiosarcoma (*n* = 1), chondrosarcoma (*n* = 1), leiomyosarcoma (*n* = 1), carcinosarcoma (*n* = 1), and malignant giant cell tumor (*n* = 1).

This study was approved by the hospital’s ethics committee, and informed consent was obtained from the families of all patients.

### 2.2 Treatment methods

**Figure 1:**
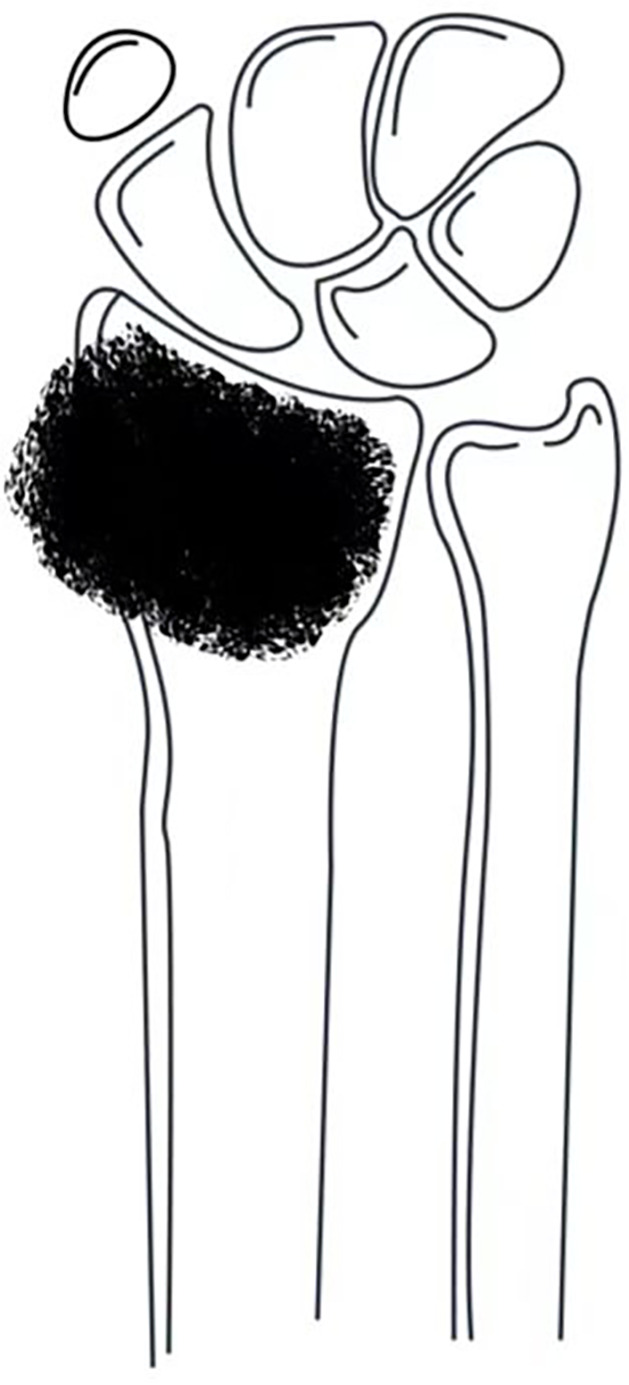
Tumor confined to the distal radius without involvement of the distal radioulnar joint or distal carpal bones.

All 11 patients underwent limb-salvage surgery. The choice of resection and reconstruction techniques was based on the extent of tumor involvement:

Tumors confined to the distal radius (**Figure 1**): Resection of the distal radius followed by joint-preserving reconstruction using either autologous fibular grafts or 3D-printed prostheses.

**Figure 2:**
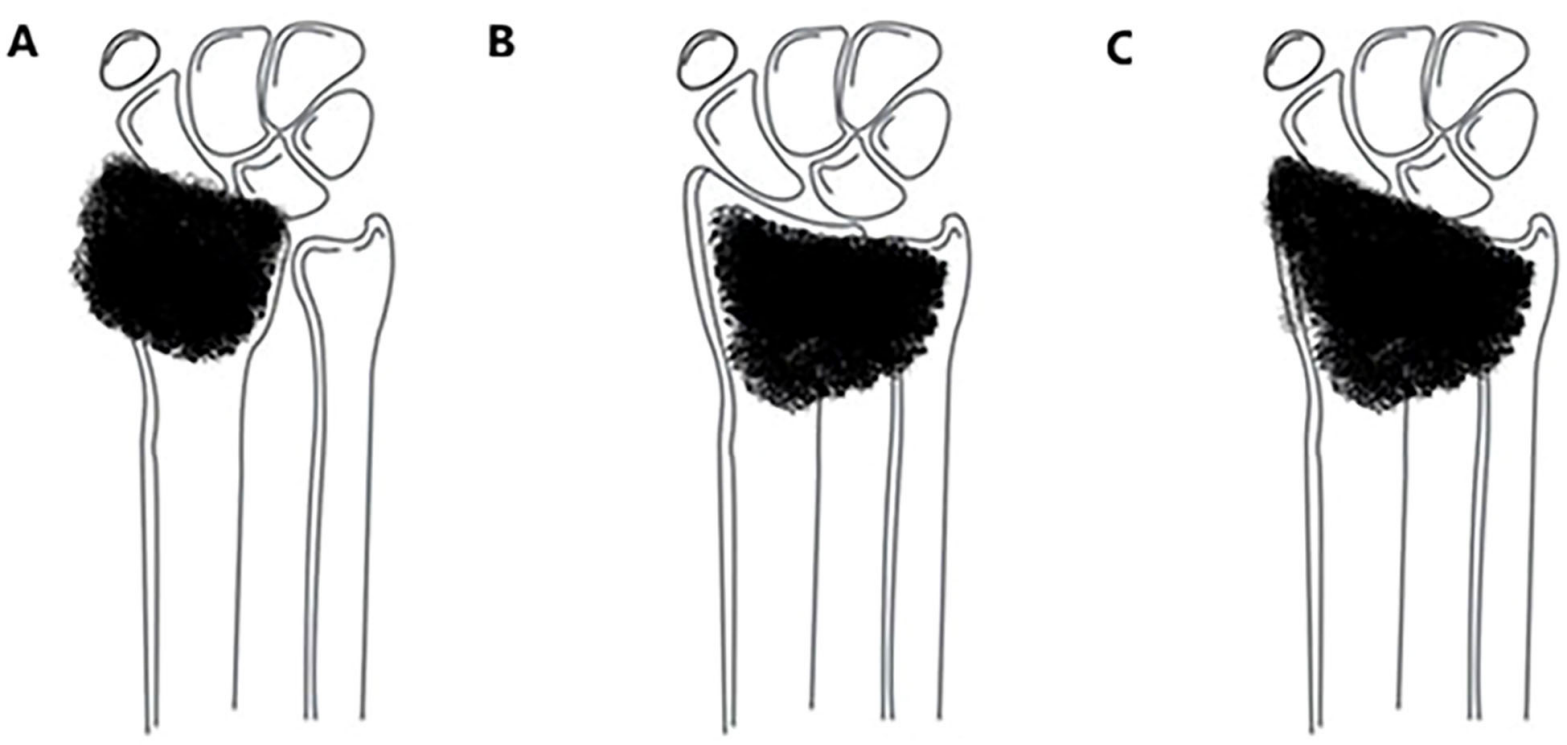
Tumor involving the distal radius with extension to the distal carpal bones and/or the distal radioulnar joint: **(A)** involving only the distal carpal bones; **(B)** involving only the distal radioulnar joint; **(C)** involving both the distal carpal bones and the distal radioulnar joint.

Tumors involving the distal radius, proximal carpal bones, and/or distal ulna (**Figure 2**): Resection of the involved structures, followed by wrist arthrodesis using either autologous fibular grafts or ipsilateral ulnar transposition with arthrodesis.

**Figure 3:**
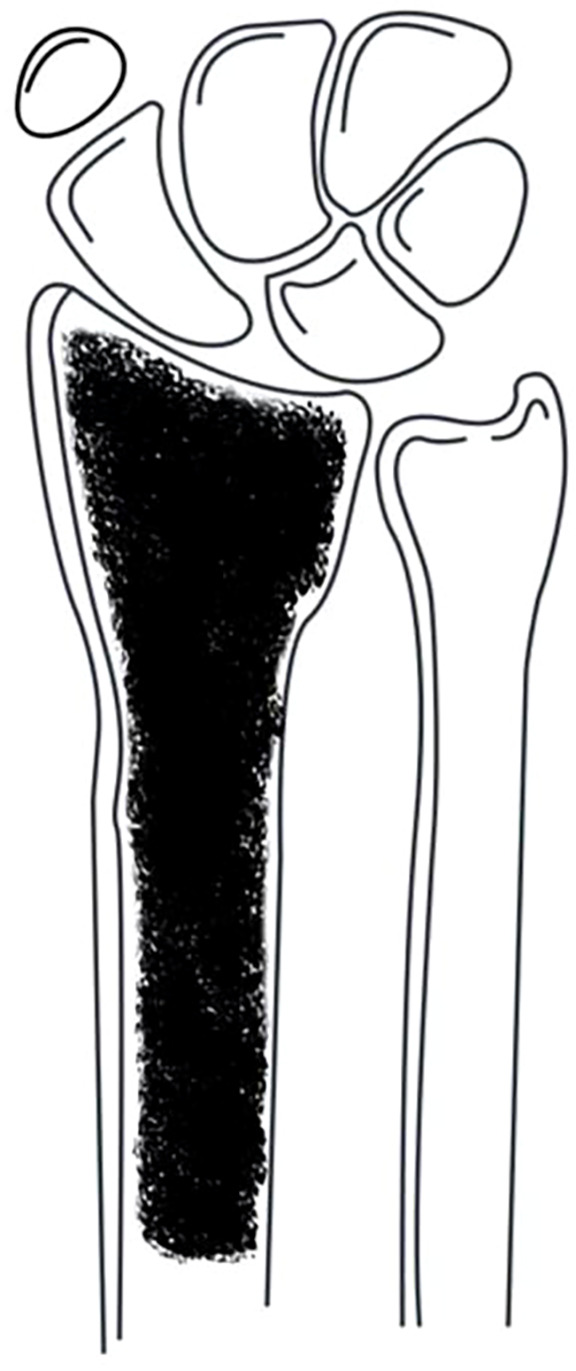
Tumor involvement extending beyond half the length of the distal radius.

Tumors involving more than half of the distal radius (**Figure 3**): Reconstruction strategies included ulnar-only fixation and wrist arthrodesis.

Additionally, three patients received adjuvant chemotherapy, while four received neoadjuvant chemotherapy.

### 2.3 Observation indicators

Postoperative data were collected through telephone interviews and outpatient follow-ups. Survival time was defined as the duration from the date of diagnosis to the date of death or the last follow-up. Follow-up assessments included verification of patient demographics (sex, age), clinical information, and surgical details, as well as monitoring for complications, recurrence, metastasis, survival status, and functional outcomes using the Musculoskeletal Tumor Society (MSTS) scoring system (6).

## 3 Results

### 3.1 Oncological prognosis

**Figure 4:**
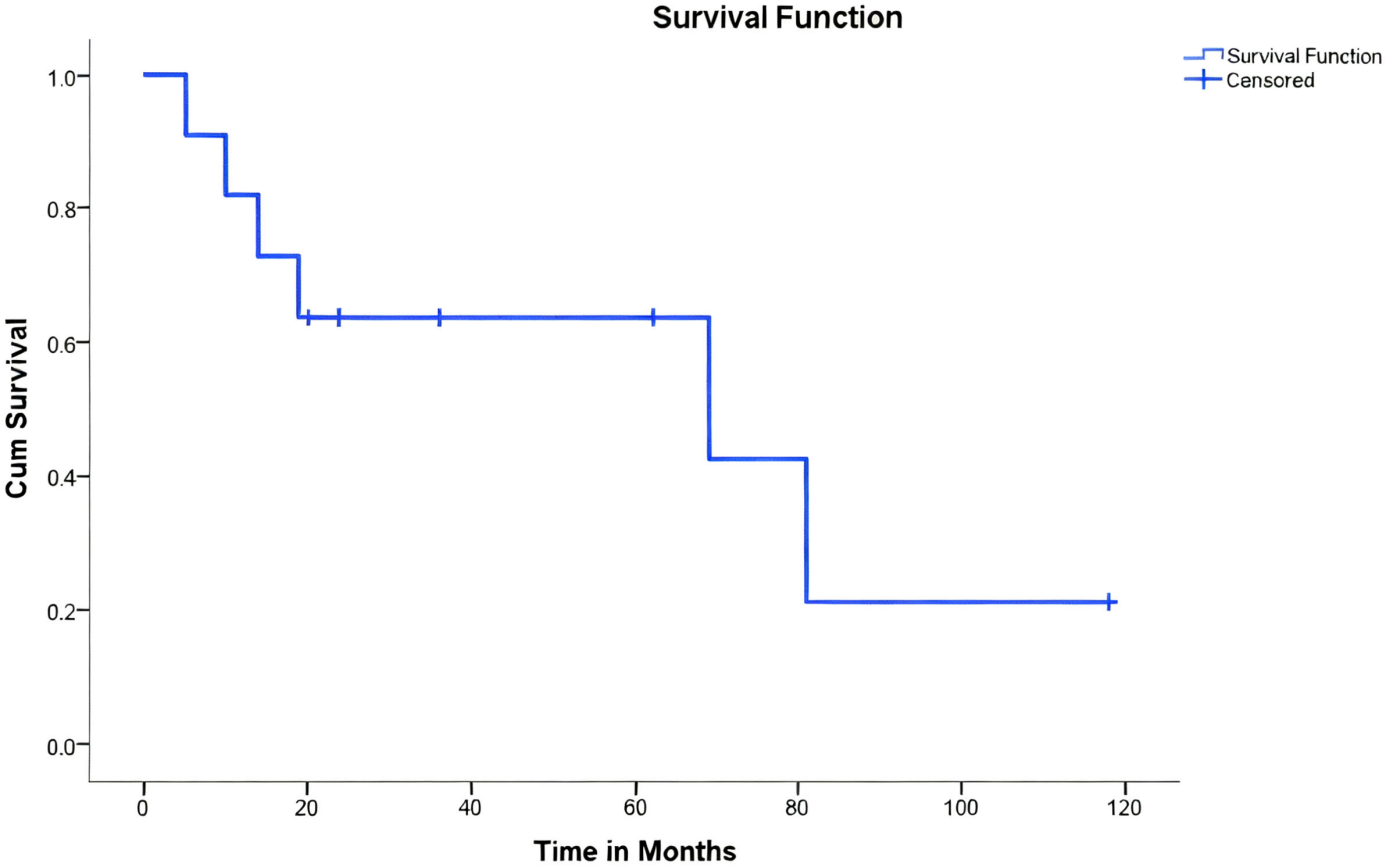
Kaplan-Meier survival analysis of 11 patients undergoing limb-salvage surgery for primary bone tumors of the radius. The Kaplan-Meier curve illustrates the overall survival probability of the patient cohort. The X-axis represents time in months post-surgery. The Y-axis represents the cumulative survival probability. A drop in the curve indicates a patient death event. Tick marks (+) on the curve represent censored data (patients who were alive at the last follow-up).

The 11 patients were followed up for periods ranging from 5 to 118 months, with an average follow-up of 41.6 months. At the final follow-up, three patients had a recurrence, six had succumbed to the disease, and five remained alive (**Figure 4**).

Among the three patients with recurrence, one developed a local soft tissue recurrence and underwent a second resection, while the other two required amputation. Among the six patients who succumbed to the disease, five died due to metastatic progression, while one patient with osteosarcoma passed away due to immunosuppression and subsequent varicella infection post-chemotherapy.

Of the five surviving patients, four had an average MSTS functional score of 26.5 (range: 24–28). One patient did not undergo MSTS assessment due to amputation.

### 3.2 Summary of resection and reconstruction methods for primary malignant tumors of the distal radius

The 11 cases of primary malignant tumors in the distal radius were treated as follows (**Table 1**):

**Table 1:** Patient data and summary of resection and reconstruction methods used (*n* = 11).

Patient	Gender	Age	Pathology	Surgery Resection	Reconstruction	Chemotherapy	Recurrence	Metastasis	Death	Follow up (Month)	MSTS Score
1	Male	30	osteosarcoma	radius+proximal wrist	Fibula Transplantation and Fusion	neoadjuvant chemotherapy	✓	✓	✓	81	/
2	Female	9	osteosarcoma	radius	fibula transplantation	neoadjuvant chemotherapy	/	/	/	118	28
3	Female	69	undifferentiated sarcoma	radius	fibula transplantation	/	✓	✓	✓	69	/
4	Female	32	Vascular Sarcoma	Radius+Ulnar+ Proximal Carpal	Fibula Transplantation and Fusion	Postoperative Adjuvant Chemotherapy	/	/	/	62	26
5	Male	20	osteosarcoma	radius	fibula transplantation	neoadjuvant chemotherapy	/	/	✓	5	/
6	Male	55	chondrosarcoma	radius	fibula transplantation	/	/	/	/	36	24
7	Male	46	leiomyosarcoma	radius+ulna+proximal carpus	ipsilateral ulna displacement and fusion	/	/	✓	✓	14	/
8	Male	66	undifferentiated sarcoma	radius+ulna	ipsilateral ulna displacement fusion surgery	postoperative adjuvant chemotherapy	/	✓	✓	19	/
9	Female	67	Malignant Giant Cell Tumor of Bone	radius	3D Printing Prosthesis Reconstruction	/	/	/	/	24	28
10	Male	74	cancer sarcoma	radius	single bone fixation+ulnar wrist fusion	adjuvant chemotherapy after surgery	/	✓	✓	10	/
11	Female	7	osteosarcoma	radius+ulna+proximal wrist	Fibula Transplantation and Fusion	neoadjuvant chemotherapy	✓	/	/	20	amputation

/: indicates absence or none; √: indicates presence; MSTS, Musculoskeletal Tumor Society.

#### 3.2.1 Isolated distal radius resection

**Figure 5:**
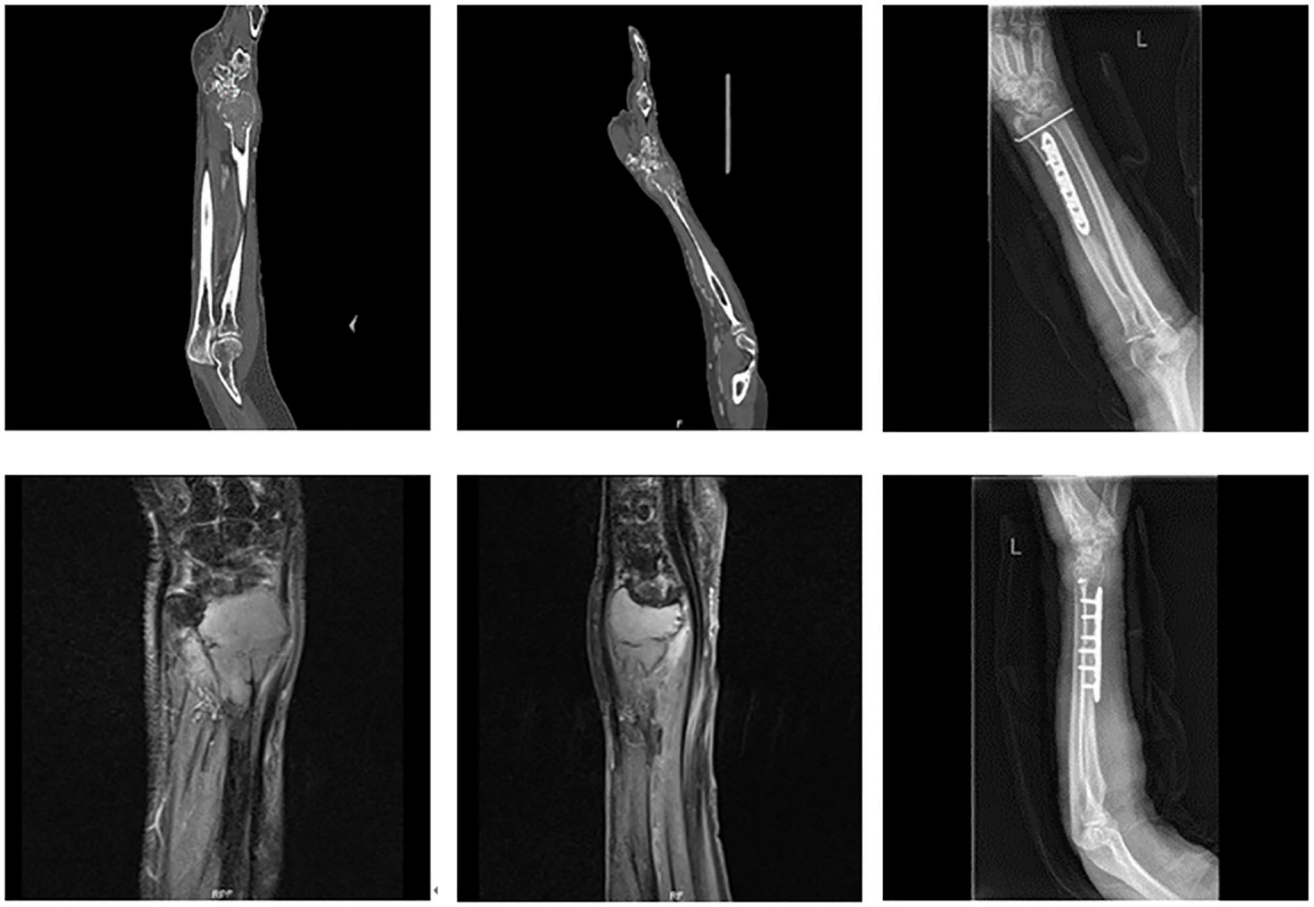
A 69-year-old female with undifferentiated sarcoma of the left distal radius. The patient underwent large segmental resection and reconstruction with an autologous fibular graft.

**Figure 6:**
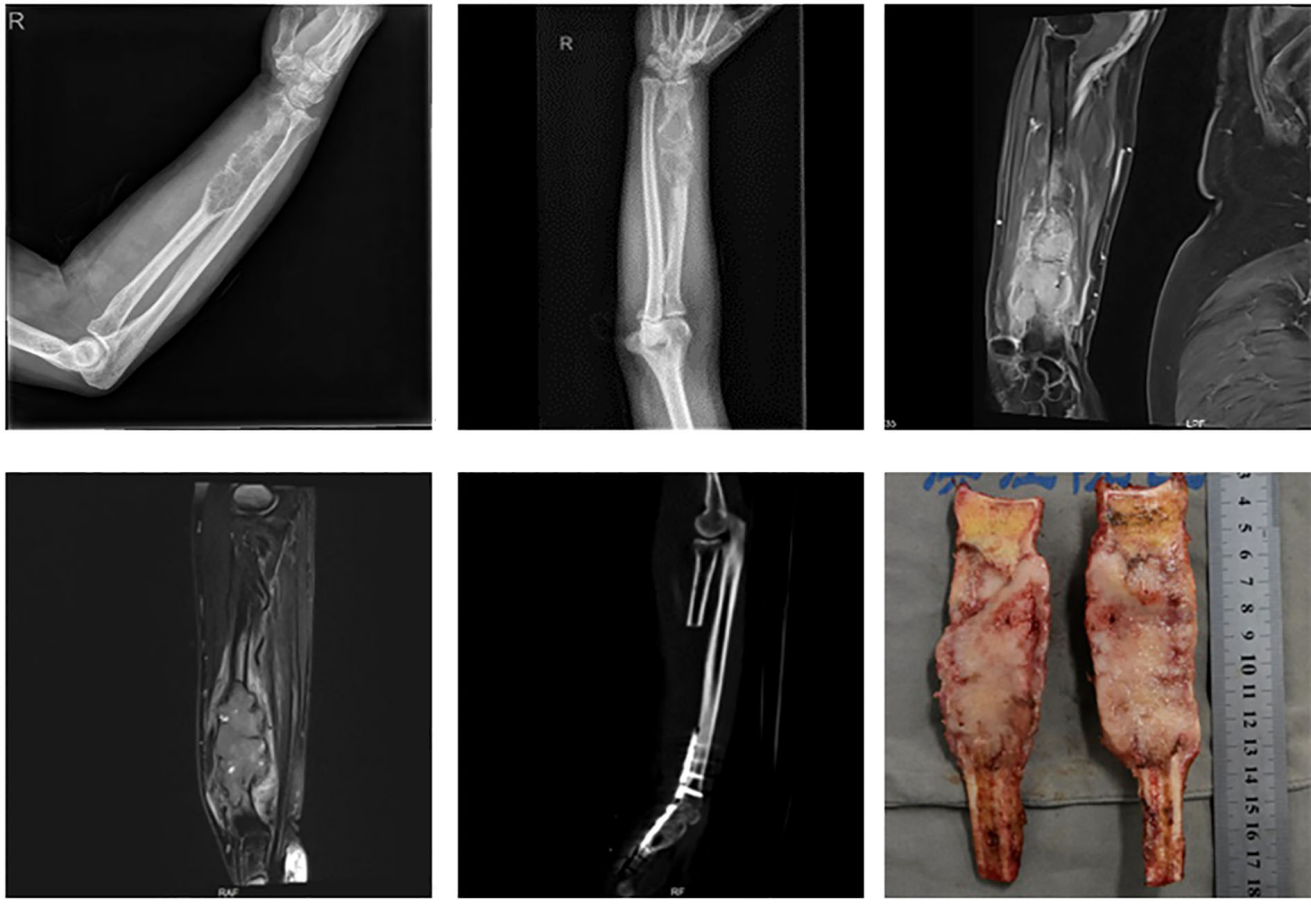
A 74-year-old male with carcinosarcoma of the distal right radius involving more than half of the bone. The patient underwent large segmental resection followed by ulnar-wrist arthrodesis.

Six cases involved isolated resection of the distal radius. Of these, five underwent joint-preserving reconstruction—four with autologous fibular grafts and one with a 3D-printed prosthesis (**Figure 5**). In one case, where tumor involvement exceeded 50% of the radial length, reconstruction was performed using ulnar-only fixation and ulnar-wrist arthrodesis (**Figure 6**).

#### 3.2.2 Resection of the distal radius and additional structures

**Figure 7:**
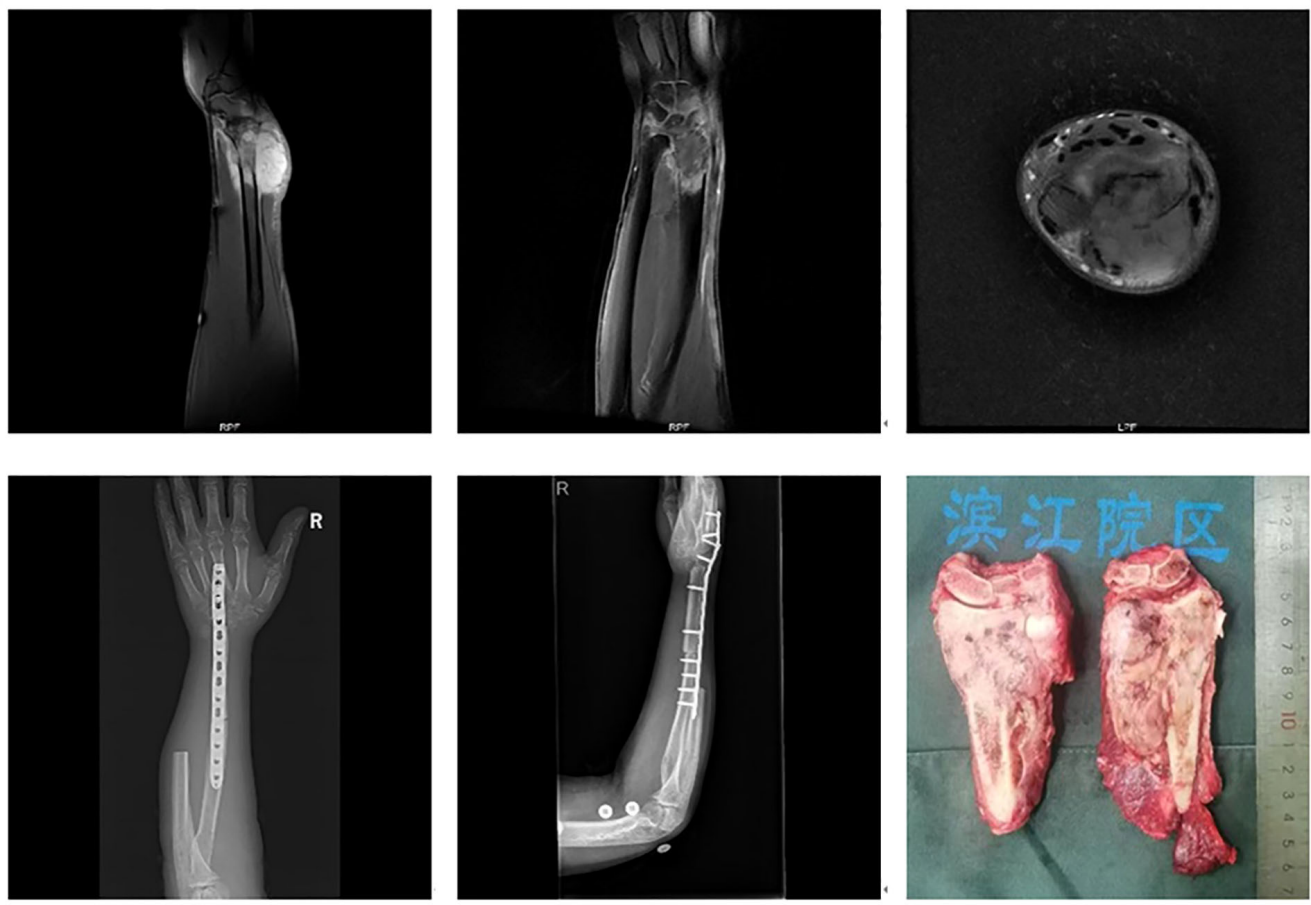
A 46-year-old male with leiomyosarcoma of the right distal radius, with tumor involvement extending to the distal radius, proximal carpal bones, and the distal ulnar-radial joint. The patient underwent resection of the distal radius, distal ulna, and proximal carpal bones, followed by ipsilateral ulnar transposition with arthrodesis.

Five cases involved extended resections: one case of distal radius and distal ulna resection, one case of distal radius and proximal carpal bone resection, and three cases involving resection of the distal radius, distal ulna, and proximal carpal bones. All cases were reconstructed with wrist arthrodesis—three using autologous fibular grafts and two using ipsilateral ulnar transposition with arthrodesis (**Figure 7**).

## 4 Discussion

Primary malignant tumors of the distal radius are rare, with only case reports available and no large series studies to provide comprehensive data. This lack of extensive research has resulted in limited understanding among surgeons regarding treatment strategies and prognosis (7–9). In this study, the oncological outcomes of 11 cases of primary malignant tumors in the distal radius were summarized. It was seen that local resection and reconstruction yielded satisfactory functional outcomes, with an average MSTS score of 26.5. However, the overall survival prognosis was poor, with six patient deaths, more than half of which were due to metastatic disease. As a non-weight-bearing bone, the distal radius often presents with no early symptoms, resulting in delayed diagnoses that allow extensive tumor invasion and an increased risk of pulmonary metastasis.

Reconstruction methods for distal radius bone defects primarily include joint-preserving techniques and wrist arthrodesis. The aim of using joint-preserving approaches is to maintain wrist function, but this may lead to complications such as joint instability, deformity, and chronic pain. Common reconstruction options include autologous fibular grafts, allografts, and prosthetic implants. Allografts, while effective, require a suitable donor and are associated with various complications such as fractures, non-union, infection, and immune rejection. Complication rates ranging from 5.9% to 26.7% have been reported, with non-union rates as high as 42.9% (10–13), which has led to a decline in their use.

Autologous fibular grafts remain a common choice for reconstruction following distal radius resection, with favorable outcomes indicated in studies (14, 15). The anatomical shape of the fibular head closely resembles that of the distal radius, facilitating effective bone healing and biological reconstruction. However, the use of autologous fibular grafts for reconstructing large bone defects following the resection of primary malignant tumors in the distal radius needs careful evaluation of the patient’s prognosis. This is particularly relevant for individuals with high-grade tumors and a poor survival outlook, as the fibular head necessitates additional surgical morbidity.

The progressive development of prosthetic implants has contributed to their increasing use in distal radius reconstruction. Although prosthetic complications, such as subluxation and aseptic loosening, have been reported in approximately 60% of cases, severe complications remain relatively rare (16). Artificial prostheses, including 3D-printed implants, are increasingly being used in reconstructive surgery due to their potential to reduce prosthesis loosening (17). In this study, autologous fibular grafts were used in four out of five cases involving isolated distal radius resection, while a 3D-printed prosthesis was used in one case. Both techniques appear suitable for cases requiring simple distal radius resection. Given the poor prognosis associated with malignant tumors of the distal radius, prosthetic solutions—especially 3D-printed implants—may become increasingly important in future reconstruction strategies.

Wrist arthrodesis techniques for distal radius reconstruction include the use of autologous iliac bone grafts, autologous fibular head grafts, ipsilateral ulnar transposition, and prosthetic arthrodesis. Arthrodesis is generally preferred in cases of joint instability, particularly when resection involves the proximal carpal bones and/or the distal ulna (18, 19). While autologous fibular head grafting is effective, it necessitates fibular harvesting, thereby increasing surgical complexity. In contrast, ipsilateral ulnar transposition eliminates the need for fibular grafting and has been associated with favorable outcomes and reduced complication rates. This method, first described by Seradge in 1982, has since been widely adopted for distal radius reconstruction (20–22).

In this study, one patient underwent resection of the distal radius and proximal carpal bones, another required distal radius and distal ulna resection, and three underwent resection of the distal radius, distal ulna, and proximal carpal bones. Autologous fibular head arthrodesis was employed in earlier cases; however, ipsilateral ulnar transposition with arthrodesis was favored in more recent cases due to its lower complication rate. Prosthetic arthrodesis was used less frequently, given its associated risks, while autologous fibular head grafts remained a viable option for high-grade tumors but required careful patient selection.

Additionally, one patient with extensive distal radius involvement (> 50% of the bone length) underwent ulnar-only fixation with wrist arthrodesis. This approach, first reported by Sayre in 1893 for pediatric radial deformities (23), has since been applied in cases of failed distal radius reconstruction (24, 25). Given the extent of bone loss in this particular case, ulnar-only fixation with wrist arthrodesis was a viable reconstructive option.

### 4.1 Limitations

The limitations of this study include the following: 1) As a retrospective study, it is subject to potential biases that may affect the accuracy of the data; 2) the small sample size may limit the generalizability of the findings; and 3) the long study period introduces variability in treatment approaches and outcome assessments.

In future research, the focus should be on expanding the sample size, refining classification criteria, and establishing standardized guidelines to enhance clinical decision-making.

### 4.2 Conclusion

Primary malignant tumors of the distal radius are rare and often associated with poor prognoses. Treatment strategies, including resection and reconstruction, should be individualized based on the extent of tumor involvement to optimize functional and oncological outcomes.

## Data Availability

The original contributions presented in the study are included in the article/supplementary material. Further inquiries can be directed to the corresponding author.
